# Med Spas: Patient Safety and Accreditation

**DOI:** 10.1093/asj/sjag085

**Published:** 2026-04-24

**Authors:** Robert Singer, Mark Jewell, Renato Saltz, Thomas Fiala

Seismic changes have reshaped the field of aesthetic surgery and cosmetic medicine over the past 2 decades. The globalization of beauty, evolving demographics, shifting cultural preferences, increasing patient expectations, and rapid technological advancements have all contributed to transforming the aesthetic marketplace. One of the most profound shifts has been the exponential growth of nonsurgical procedures, which now far exceed traditional surgical interventions. This has led to the growth of med spas.^1^

The world of med spas today is essentially like the Wild West of retail medicine. With little or no regulatory oversight, there is the potential for lack of appropriate medical supervision, lack of appropriate protocols even for advanced techniques, and poor preparation for treating emergencies and complications when they do occur. Unfortunately, patient safety has taken a back seat.

The number of med spas in the United States has exploded, with an estimated 13,000 nationwide by the end of 2026. Today there are almost as many med spas as there are McDonald's locations in the United States, and in some states, like Florida, med spas outnumber the burger giant by 2.5 to 1.^2^ The growth has outpaced regulations. Patients expect and deserve safe, high-quality care in every setting, including the modern med spa, but unfortunately they cannot tell which spas are safe.

The working definition of a med spa is a freestanding location offering medical procedures that are usually performed in a physician's office, generally done for appearance or well-being in a spa-like atmosphere. The med spa offers a range of services focused on beauty and rejuvenation. It is not part of a physician's office, adjacent suite, or office surgery suite, and it is not regulated under the same rules as they are.

There may or may not be a supervising physician on site or available, who is appropriately trained in all services offered in the med spa. Medical spas are different from how they have been in the past; many now involve wellness, hormonal treatments, intravenous therapy, GLP-1 agonists, and gynecologic procedures (sexual wellness) (Table 1). The term “med spa” does not imply any regulatory monitoring. In most cases it translates to no oversight, no standards, and in some cases no accountability.^3^

**Table 1.** Today's Med Spa Spectrum

Spectrum of Today's Medspas
More than just “botulinum toxin and fillers”
Aesthetic (botulinum toxin, fillers, lasers, peels, facials, nonsurgical fat reduction)
Hormone management
Sexual and intimate wellness
Weight loss, with GLP-1 (glucagon-like peptide-1 receptor) agonists
Intravenous therapy
Functional and integrative medicine
Not just run by plastic surgeons and other “core 4” physicians anymore
Family practitioners, OB-GYNs, chiropractors, RNs, APRNs, PAs, aestheticians, “laser technicians,” MAs, and nonmedical personnel now run many med spas, with increasing corporate ownership and franchising

APRN, advanced practice registered nurse; MA, medical assistant; OB-GYN, obstetrician/gynecologist; PA, physician’s assistant; RN, registered nurse.

The ease of opening a storefront and labeling it as a med spa has blurred things for our patients. Even physician-run or physician-directed med spas are perceived like many others, often indistinguishable from the pop-up med spa across town that just bought a laser through Instagram. Patients start second-guessing legitimacy, and it is challenging to determine who is qualified.^4^

It’s important to understand that not all med spas are equal. Although some provide appropriate, safe procedures, others are operated by doctors from unrelated specialties who may have little or no training in cosmetic procedures or their management, safety, and risk for complications. Others may be run by nonphysicians, such as nurses or physician's assistants, who typically receive far less medical training and may not have an in-depth knowledge of the anatomy involved in many of these treatments.^5^ Although most med spa treatments are considered low risk, they still involve one’s health and appearance; they are medical treatments and should be treated as such.

Things to consider include the following: Who ‘s actually overseeing the treatment? Do they offer a consultation? What services are available? It is important that the med spa follows state laws with regard to who oversees and provides the procedure, but most states have little or no guidelines for med spas. How qualified are the providers? Is the facility clean and safe? What technology and products do they use? Is there transparency in pricing? Are the treatments customized? What is the aftercare? What happens in the case of a complication or an emergency?

## REGULATORY AND SAFETY CONCERNS

In most states, med spas are unregulated. This has resulted in an increased number of patients harmed, with an increasing amount of press coverage from national news organizations and consumer-facing magazines around this issue.^3,6-10^ There have been reports of HIV, hepatitis, and deaths in med spas.^11-14^

A survey by the American Society for Dermatologic Surgery found that burns, discoloration, and misplacement of product were the most commonly reported complications at aesthetic medical spas, with fillers, intense pulsed light (IPL), and laser hair removal being the treatments most frequently resulting in sequelae. Also, when compared to physician-based practices, medical spas have been rated as inferior in both safety and outcomes.^15^

A large public survey demonstrated that total complication rates were numerically higher at medical spas compared to physician offices, with minimally invasive skin tightening and nonsurgical fat reduction having statistically significant increases in complication rates at medical spas.^16^ Patient surveys revealed that more burns and discoloration occurred in the nonphysician-treated group, particularly in med spas.^17-20^

Medical spas now outnumber physician-based cosmetic practices in the majority of large US cities.^21^ We concur with the American Academy of Dermatology Association’s statement, “While cosmetic treatments may appear simple, performing them safely requires an in-depth understanding of the structure and function of the skin. This advanced expertise allows them (Board-Certified Dermatologists) to minimize risks and effectively manage any complications that may arise.”^22^ Of course, plastic surgeons also share this training and knowledge base.^23-27^

Because of the need for patient safety and the lack of oversight in most states, the senior author of this article started a task force to develop criteria for accreditation of med spas. There was input sought from the “core” group of physicians, plastic surgeons, facial plastic surgeons, dermatologists, and maxillofacial surgeons, as well as obstetric and gynecologic physicians, midlevel providers, practice consultants, and ancillary personnel (Table 2). Rather than issuing a prescriptive set of rules, the group worked to develop a certification process based on reasonable and rational standards. The group was careful to obtain input from a variety of clinicians in different fields, to ensure that the standards were accurate and unbiased.

**Table 2.** Med Spa Task Force Work Group

We Acknowledge the Assistance of the Following Individuals
Chair: Robert Singer Plastic surgeons: Thomas Fiala, Mark Jewell, Renato Saltz, Jennifer Walden, Brad Calobrace, David Zargaran OB-GYNs: Red Alinson, Wesley Brady Dermatologist: Leslie Bauman Practice consultants: Yvonne Dellos, Christy Perry, Terri Ross Media: Mike Glacier (*NewBeauty*) Industry: Tiphany Hall, Judy Kozlicki Advanced practice providers (APPs): Marisa Faircloth, Julie Frost, Laura Matajasich QUAD A: Patty Chmielewski, Monda Shaver, Thomas Terranova

OB-GYN, obstetrician/gynecologist; QUAD A, American Association for Accreditation of Ambulatory Surgery Facilities.

There is nothing in the literature about how to improve patient safety in med spas. Our manuscript highlights the American Association for Accreditation of Ambulatory Surgery Facilities (QUAD A) comprehensive approach to patient safety through facility accreditation, staff training, and procedural designation, according to scope of practice. This is different from other entities that suggest awarding a certificate without appropriate standards, protocols, or an actual facility inspection.

## WHY ACCREDIT A MED SPA?

External quality evaluation for certification can enhance trust, transparency, and patient safety.

Certified med spas can stand out, giving patients added confidence in their choice of facilities, essentially like the “Good Housekeeping Seal”. The certification process can bring much-needed structure, oversight, and credibility to the rapidly expanding arena of med spas and wellness settings, leading to the consistent delivery of safer, high-quality care and improved patient safety. Existing state regulations also are considered. Facilities will have to meet the higher standards from accrediting boards and individual states.

The spa accreditation task force worked over the last year to develop the guidelines, which were then sent to QUAD A. The QUAD A Standards Committee evaluated the guidelines and approved the process for accreditation of med spas, which was implemented beginning in March 2026 on a national basis.^28^

It is important to have clear, consistent standards that patients can trust. The accreditation of medical spas by QUAD A, the “gold standard” of accreditation, is an opportunity to improve patient safety, which is one of the core objectives of QUAD A. It is a similar arrangement to QUAD A's Office Surgery Center accreditation, which has proven its value for patient safety, although the new standards have been completely rewritten so that they are relevant and applicable to the medical spa setting.^29,30^

QUAD A's nonsurgical aesthetic and wellness (med spa) accreditation program provides thorough assessments based on rigorous standards that help facilities meet patients’ expectations and position themselves as trusted providers in a growing field. One of the key aspects is the requirement of training and experience for those providing the procedures, as well as those who are supervising and delegating the procedures.

## ACCREDITATION PROCESS

On-site or virtual inspection of the physical plant by trained QUAD A inspectors is performed on a 3-year cycle. Interviews are performed with the medical director regarding training, responsibility, availability, oversight, and key personnel. Accreditation will offer trust and patient safety. Guidelines cover the clinical environment, safety procedures, records, compilation of complications, risk stratification, staff qualifications, which procedures can be performed by whom, and which tier can provide them. Safety standards, education of providers, oversight, sterility, infection prevention, and review of equipment are addressed. Total (100%) compliance with med spa standards is required to pass.

As can be seen, this program comprises much more than sending in a request for something to display on the wall, paying a fee, and automatically obtaining a certificate. It requires site inspection and thoughtful compliance with 100% of the guidelines. Accreditation helps med spas meet patient expectations by providing an independent, structured framework that aligns the med spa with recognized safety expectations.^31^

There are 4 main areas of med spa standards.

1. General: facility layout and environmental standards; anesthesia options (topical, local); truth in marketing; and a Patient Bill of Rights.
2. Safety: facility safety manual and handling of hazardous agents; fire safety standards; staff safety standards; required emergency equipment and medications; and emergency protocols for filler emergencies and laser and chemical peel treatments.
3. Training and education: medical director requirements; training and education of all personnel; and personnel files.
4. Other clinical standards: infection control; clinical record requirements; and mandated reporting of complications.

## WHICH PROCEDURES? BY WHOM?

Based on published literature, a risk-stratification system was adopted for common med spa procedures, and the working group had extensive discussions about which category the procedures belonged in, who could reasonably perform them, and the training and experience they needed before being relatively safe, especially if they had not completed an Accreditation Council for Graduate Medical Education (ACGME)–approved residency in a relevant field. There are 4 categories: green, yellow, orange, and red. Note that traditional surgical procedures, typically performed in an office surgery center, are in the red category, because it was felt that these were not suitable for the med spa environment.

Superficial or epidermis procedures that are very low risk and can be performed by an aesthetician or medical assistant are in the green category. Examples include a traditional facial or lymphatic massage.

Botulinum toxin and dermal fillers placed within the head and neck region are in the yellow category. If permitted by state regulations, nurses can perform these. Otherwise, they are to be done by midlevel providers and physicians. Some procedures, regarded as low to moderate risk, such as nonablative laser treatment, require additional training, even though they are in the yellow category.

Middle or deep dermis procedures are considered more moderate risk (orange category) and are to be performed by a midlevel provider or physician only. This category includes ablative laser treatment, thread-lift procedures, and midlevel chemical peels. Treatments of the male and female genitalia are in this category, and these require additional specific training, which is outlined in the standards.

## ROLE OF MEDICAL DIRECTOR

One of the other big changes in this program involves the role of the medical director of the med spa. The medical director is now a key figure, with multiple responsibilities and direct involvement. It is not a figurehead position. Typically, the medical director is a physician with an active license in the state where the med spa is located and is available by telemedicine for immediate consultation. In states in which advanced practice registered nurses (APRNs) have been granted independent practice rights by their state legislature, they also may serve as a medical director. In either case, the medical director has full legal responsibilities for the operations and policies of the med spa. Following the principle of “you cannot supervise what you do not know,” the medical director must have the education, training, and experience to competently perform, administer, delegate, and supervise all procedures performed at the med spa. Additionally, the medical director is to be clearly identified in the med spa's marketing materials and on websites and social media, including their specific training and board certifications by the American Board of Medical Specialties (ABMS). A medical director is limited to supervising 3 locations.

## BENEFITS

QUAD A's med spa accreditation program gives facilities a clear, credible framework to demonstrate their commitment to patient safety, responsible practice, and operational excellence. With this, med spas have the opportunity to adopt structured, evidenced-based standards that support safer, more consistent care. The program provides the ability to differentiate top-quality spas from the rest and hopefully encourage other med spas to improve safety and seek accreditation.

Very few states have any regulations, nor do they have the resources or workforce to inspect the multitude of med spas. It is hoped that states will adopt the concept of accreditation of med spas by QUAD A and mandate accreditation for patient safety.

To be successful, the program will require a public marketing campaign and will take time to gain momentum. The authors feel that it is important that plastic surgeons take the lead in the effort and regain leadership in this area. Ultimately, it will improve patient safety and the image of spas.

To help protect the public, the authors encourage all providers to reach out to their state medical boards and legislators to mandate accreditation standards for med spas.
